# Effectiveness of a multiple-strategy community intervention to reduce maternal and child health inequalities in Haryana, North India: a mixed-methods study protocol

**DOI:** 10.3402/gha.v8.25987

**Published:** 2015-02-10

**Authors:** Madhu Gupta, Federica Angeli, Onno C. P. van Schayck, Hans Bosma

**Affiliations:** 1 Department of Community Medicine, School of Public Health, Post Graduate Institute of Medical Education and Research, Chandigarh, India; 2 Department of Health Services Research, CAPHRI, Maastricht University, Maastricht, The Netherlands; 3 Department of Family Practice, CAPHRI, Maastricht University, Maastricht, The Netherlands; 4 Department of Social Medicine, CAPHRI, Maastricht University, Maastricht, The Netherlands

**Keywords:** National Rural Health Mission, India, health inequalities, mixed-methods approach, maternal health, child health, health indicators

## Abstract

**Background:**

A multiple-strategy community intervention, known as National Rural Health Mission (NRHM), launched in India to improve the availability of and access to better-quality healthcare, especially for rural, poor mothers and children. The final goal of the intervention is to reduce maternal and child health inequalities across geographical areas, socioeconomic status groups, and sex of the child. Extensive, in-depth research is necessary to assess the effectiveness of NRHM, on multiple outcome dimensions. This paper presents the design of a new study, able to overcome the shortcomings of previous research.

**Objective:**

To propose a comprehensive, methodologically sound protocol to assess the extent of implementation and the effectiveness of NRHM measures to improve maternal and child health outcomes and reduce maternal and child health inequalities.

**Design:**

A mixed-methods approach (quantitative and qualitative) is proposed for this study in Haryana, a state in North India. NRHM's health sector plans included health system strengthening, specific maternal and child healthcare strategies, and communitization. Mission documents and reports on progress, financial monitoring, and common and joint review will be reviewed in-depth to assess the extent of the implementation of plans. Data on maternal and child health indicators will be obtained from demographic health surveys held before, during, and after the implementation of the first phase of the NRHM (2005–2012) and compared over time. Differences in maternal and child health indicators will be used to measure maternal and child health inequalities; these will be compared pre- and post-NRHM. Focus group discussions (FGDs) with service providers and in-depth interviews with program managers, community representatives, and mothers will be conducted until data saturation is achieved, in two districts of Haryana. Using Nvivo software, an inductive qualitative content analysis will be performed to search for the broader themes across the interviews and FGDs. Ethical approval was obtained from the Ethics Committee of the Post Graduate Institute of Medical Education and Research.

Achieving millennium development goals 4 and 5 – that is, reducing mortality among children aged ≤5 years by two-thirds and maternal mortality by three quarters between 1990 and 2015 – is among the highest priorities on India's national health agenda (1). Between 2005 and 2012, India's total spending on health increased from 0.9% to nearly 2% of gross domestic product, but maternal and child health indicators have not improved correspondingly (2).

## Problem statement

The maternal mortality rate (MMR) is still as high as 178 maternal deaths per hundred thousand live births (3) and the infant mortality rate (IMR) is 42 infant deaths per thousand live births (4). There is geographical inequality in maternal and child health outcomes. For example, IMR is higher in rural as compared to urban areas (48 vs. 28 deaths per thousand live births) (4). Large geographical and socioeconomic inequalities in maternal and child health status and access to health services continue to persist in India and have even widened across states, between rural and urban areas, and within communities (5). Singh et al. reported inequality regarding advice during the antenatal period and the coverage of essential postnatal care, which is provided disproportionately more frequently among the rich (6, 7). Nayar reported maternal and child health inequalities across different caste groups [scheduled castes (SC), scheduled tribes (ST), other backward castes (OBC), and general castes] in India. SC, ST, and OBC (lower castes) represent communities belonging to lower socioeconomic groups with a poor maternal and child health status as compared to higher castes (8). Pathak et al. reported a disproportionately concentrated malnutrition burden among poor children and slow changes in child malnutrition in India during 1992–2006, coupled with a concomitant rise in economic inequalities (9). Pradhan et al. reported that poor household economic status (46%), mother's illiteracy (35%), and rural residence (15%) explained 96% of the total socioeconomic inequalities in child survival at the national level (10). Sex inequality among children is another area of concern, especially among states in North India, involving a strong preference for sons, widespread female feticide, and declining sex ratio at birth (11). Sex disparity in immunization programs favoring males has been reported in urban areas, developed states, and Muslim communities in India (12). Better healthcare-seeking behaviors of caregivers for sick male children as compared to sick female children further add to the sex-related health inequalities.

This persistence of maternal and child health inequalities highlights the need to assess how the existing national health programs or policies on maternal and child health are being implemented. Simultaneously, it indicates the need for studies on the effectiveness of these programs, as these are highly resource intensive. Such assessments can inform policy makers in resource-constrained countries such as India on ways to improve the policy or implementation strategy of these interventions.

## Study population

Studying maternal and child health inequalities in Haryana is worthwhile as it is representative of other states in North India with similar socioeconomic development and sociocultural factors, such as the preference to have sons, female feticide, lower sex ratios, and lower social status of women. At the same time, Haryana represents a unique context by being a prosperous state with a rising economy but with unequal distribution of resources, which has led to wide intra-state and inter-district differences in terms of provision of basic infrastructure such as water, roads, schools, hospitals, and so on. Despite being one of the richer states, reporting the highest per capita income in the country at Rs 109,064 (USD 1947.6) during 2012–2013, maternal and child health indicators are not the best in the country (13). Although the MMR has declined from 176 (for the year 1999–2001) (14) to 146 deaths per one hundred thousand live births (for the year 2010–12) (3), it still lags behind the goal of reducing it to below 100 by 2015 (1). There are marked geographical differences in maternal and child health: the IMR is higher in rural areas (46 per thousand live births) compared to urban areas (33 per thousand live births) (4). The child sex ratio at birth declined from 964 in the 2001 census to 830 per thousand males in the 2011 census (12). There is a clear problem of female feticide and poor health-seeking behavior for daughters (15–18). All this provides us with an excellent opportunity to study inequalities in this state.

## Current and past interventions/current state, new interventions

Past interventions to improve maternal and child health were initially implemented as vertical programs, such as the Family Welfare Program (1952), Acute Diarrheal Disease Control Program (1978), Acute Respiratory Infections Control Program (1978), and Universal Immunization Program (1985). These initiatives were later merged, initially as the Safe Childhood and Safe Motherhood Program (CSSM, 1992) and then as the Reproductive and Child Health Program (RCH I, 1997–2005), as it was realized that improving maternal health is imperative to improving child health (19). However, the main objective in these earlier programs was to improve the maternal and child health indicators and increase their survival, and not much emphasis was put on reducing inequalities. Realizing this gap in implementation, a national multiple-strategy community intervention was launched, known as the National Rural Health Mission (NRHM), by the Ministry of Health and Family Welfare of the Government of India [started during 2005 in the 11th health plan (2005–2012), and continued in the 12th health plan (2012–2017)] with the aim of reducing health inequalities by improving the availability of and access to better-quality healthcare, especially for people residing in rural areas (to reduce geographical inequality), for the poor (to reduce socioeconomic inequality), and for women and children (to reduce sex inequality) (20). NRHM's health sector plans included health system strengthening, specific maternal and child healthcare strategies/schemes (RCH-II), and communitization (delegating powers to and empowering the community to monitor the healthcare delivery system) (21). Details of these plans are given in Supplementary files. Briefly, health system strengthening involved making available mobile medical units (MMUs) and patient transport services, strengthening the health infrastructure, providing free drugs and logistics, and providing telemedicine facilities. Maternal and child health schemes included cash incentives for hospital deliveries, free delivery services for pregnant women, treatment of neonatal illnesses in hospitals, reimbursements of travel cost to hospitals, and appointing Accredited Social Health Activists (ASHAs) to promote the access to improved healthcare at household level in villages. The intention was to reduce the IMR to 30/1,000 live births, MMR to 1/1,000 live births, and the total fertility rate to 2.1 by 2012. It was further realized that NRHM strategies were not covering the urban poor, whose condition was even worse than that of the rural population. Hence, NRHM was renamed National Health Mission (2012), and now covers the slum population as well.

## Previous assessments and their strengths/weaknesses

The planning commission of India had the NRHM schemes evaluated in seven states (Uttar Pradesh, Madhya Pradesh, Jharkhand, Orissa, Assam, Jammu and Kashmir, and Tamil Nadu) during the 4th year of its implementation (2009–10) and assessed the availability, adequacy, and utilization of maternal and child health services (22). They conducted cross-sectional surveys, focus group discussions (FGDs), and in-depth interviews with stakeholders. They observed some improvements in the availability and utilization of maternal and child health services in rural areas, and recommended further strengthening of health facilities. The strength of their study lies in the inclusion of a qualitative assessment of the program that gave insight into the implementation process. However, their evaluation was limited by the lack of assessment of the extent of implementation of NRHM schemes, including budget sanctioned and spent on NRHM schemes, the lack of comparison of results with the situation before the implementation of the NRHM, the lack of measurement of maternal and child health inequalities, and the lack of interpretation of quantitative data and qualitative data by a mixed-methods approach. Because NRHM had 2 more years to go at the time of the planning commission's evaluation, it represented a mid-term evaluation. The present study intends to overcome the above limitations by assessing the extent of implementation of NRHM schemes in the maternal and child health care sector, including the budgetary outlays for maternal and child health schemes through the NRHM period (2005–12) and its effectiveness, by comparing the situation before, during, and after the implementation of the NRHM using a mixed-methods approach. In another study by Mukherjee et al., 100 rural doctors from the states of Orissa, Assam, Jharkhand, and Chhattisgarh were interviewed to analyze the effectiveness of the NRHM in improving the availability and accessibility of health services in rural areas. They concluded that it was not 100% effective and there were inefficiencies in terms of infrastructure and manpower (23). Earlier surveys did report on the effectiveness of health services, but none reported on maternal and child health inequalities. Also, no previous study has been conducted in Haryana state. State-specific information is necessary, as each state is different, having its own unique cultural, social, and demographic backgrounds and problems. Because the causes of maternal and child health inequalities vary across states, solutions to bridge the gaps thus have to be tailor-made (24, 25).

It is against this background that the present mixed-methods study was designed, to quantify the extent of implementation of NRHM's maternal and child health-related plans in the healthcare sector, to quantify NRHM's effectiveness in terms of reducing geographical, socioeconomic, and sex inequalities and improving the overall maternal and child health outcomes, as well as to qualitatively ascertain the extent to which maternal and child health strategies in the NRHM were implemented and were effective in tackling the inequalities and outcomes, and to formulate evidence-based recommendations for bridging the health inequalities in Haryana.

## Study design

A mixed-methods approach will be used in this study, involving a partially mixed sequential equal status design in terms of the Leech classification (26). Partially mixed design implies that mixing of qualitative (QUAL) and quantitative (QUAN) data will be done at interpretation level (i.e. the quantitative data will be linked to and explained by qualitative results); sequential means that the qualitative data will be collected after the quantitative data collection; and equal status denotes that both qualitative and quantitative data will be given the same importance at the time of interpretation. The quantitative part of the mixed-method study will reveal the trends and differences in rates for maternal and child health outcomes and inequalities before, during, and after NRHM implementation, whereas the qualitative study will provide explanations for these findings, which will be used to formulate evidence-based recommendations for implementing the program in a more effective way, so as to achieve the intended maternal and child health goals.

## Setting

This study will be done in Haryana. A state is divided into many administrative districts, which include several administrative blocks. The chief medical officer (CMO) is the overall person in charge of implementing national health programs at the district level. There are several program officers, one for each program, who report to the CMO. There is a three-tier system of healthcare infrastructure in each district: at grass-root level, there is a subcenter catering to a population of 5,000, a primary health center (PHC) catering to a population of 30,000, and a community health center (CHC) catering to a population of 100,000. Above the CHC (block) level, there is either a sub-district or district hospital. A doctor is available at the PHC level and above. At the sub-health center, an auxiliary nurse midwife (ANM) is responsible for implementing maternal and child health programs. She is assisted by an ASHA (6) and a child care volunteer called anganwadi worker for each village or population of 1,000 (9). Community groups include Panchayati Raj Institutions, village health committees, and self-help groups in villages (27, 28).

**Fig. 1:**
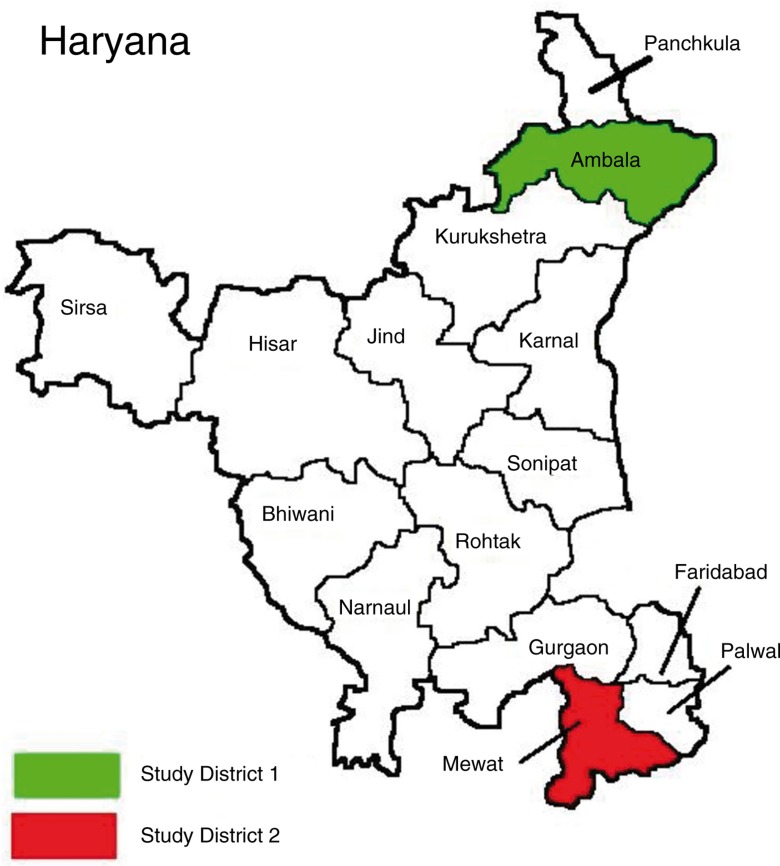
Map of Haryana showing districts selected for qualitative study, 2013.

Haryana has 21 districts, and has a population of 25,353,081 (70% rural), a birth rate of 21.6, and a mortality rate of 6.4 deaths per thousand mid-year population (4, 10). For the qualitative study, we will select a well-performing (Ambala) and a poorly performing district (Mewat) of Haryana. This selection will allow us to obtain a better contextual understanding of two extreme situations and to learn which scheme works better in a particular situation, by exploring the perceptions and beliefs of service providers, community representatives, and mothers regarding the implementation status and effectiveness of NRHM's maternal and child health schemes (Fig. 1). Criteria for labeling the district as well or less well performing are based on the District Level Household Survey 3 (DLHS-3, 2007–08) (29). Maternal and child health indicators for comparison included age at marriage below 18 years (3% in Ambala vs. 43% in Mewat), teenage pregnancies (0.9% in Ambala vs. 9.3% in Mewat), availability of antenatal care (83% in Ambala vs. 53% in Mewat), institutional births (55% in Ambala vs. 15% in Mewat), availability of postnatal care (70% vs. 34%), and fully immunized children (92% Ambala vs. 20% Mewat). About 51% of the population of Ambala has a high standard of living index, compared to 11% in Mewat. Although differing in the above characteristics, the Ambala and Mewat districts have a similar population size (Ambala 1,128,350; Mewat 1,089,263) and density (Ambala 717; Mewat 723 per square km).

## Data collection and analyses

### Quantitative study

#### Extent of implementation of NRHM plans

Information will be obtained via an in-depth review of the NRHM mission document (21), the progress reports of the NRHM, approved state program implementation plans, and financial monitoring reports, in order to obtain information on budgets approved and spent for each activity planned in the financial years 2005–2012. Independent evaluation reports, such as those by the Common Review Mission (CRM) (30) and Joint Review Mission (31) will also be reviewed. CRM reports are an important component of the overall monitoring and evaluation framework envisaged in the NRHM implementation framework. The CRM undertakes spot appraisal of the health system and reflects on the success of the strategies and policies with the aim of identifying the need for potential mid-course corrections in implementation. Seven such reviews have been conducted so far, and Haryana state was covered in the third, fifth, and seventh CRMs. Data pertaining to maternal and child health indicators in these documents will be recorded on a predesigned form to avoid selection bias. This will prevent rejection or acceptance of ‘bad’ data on arbitrary grounds instead of according to previously stated criteria as listed in predesigned form.

#### Effectiveness of NRHM plans

Information on the status of maternal and child health indicators will be obtained from the National Family Health Survey (2005–06) (32), the DLHS [DLHS 2 (2002–2003), the DLHS 3 (2007–2008), and the DLHS 4 (2012–2013)] (29). DLHS 2 represents the situation before, DLHS 3 during, and DLHS 4 after NRHM implementation. These surveys provide consistent and reliable estimates of fertility, mortality, family planning, utilization of maternal and child healthcare services, and other related indicators at both the national and state levels. MMRs and IMRs at the Haryana state level will be obtained from the Sample Registration System (3, 4).

#### Variables

Implementation variables include NRHM health sector measures (Table 1), whereas sociodemographic variables include wealth index, education, caste, and religion, as available from demographic surveys. Outcome variables for this study are listed in Table 2. These include maternal and child health indicators, maternal and child health inequalities across the socioeconomic, geographical; and sex gradients; and indicators on the access/availability of maternal and child health services.

**Table 1:** Implementation status of NRHM health sector plans

NRHM Plans (independent variables)	Implementation status		

	Full	Partial	None
Health system strengthening			
Mobile medical units with access to hard-to-reach areas	–	–	–
Patient transport service/referral services	–	–	–
Infrastructure development and strengthening: construction of new healthcare facilities for universal access to primary healthcare as per the norms, and strengthening of existing facilities as per Indian Public Health Standards (IPHS)	–	–	–
Human resources: availability of additional nurses, doctors, specialists, ANMs, and administrative staff on a contractual basis	–	–	–
Drugs and logistics (free essential medicines at all healthcare facilities)	–	–	–
Telemedicine	–	–	–
Communitization			
Accredited Social Health Activist (ASHA)	–	–	–
Village health and sanitation committees	–	–	–
Village health and nutrition days	–	–	–
*Rogi Kalyan Samities* (patient welfare committees in the hospitals with members also deriving from the community)	–	–	–
Maternal healthcare strategies			
*JSY – Janani Suraksha Yojna*	–	–	–
*JSSK – Janani Shishu Suraksha Karyakaram*	–	–	–
Increased number of delivery points with provision of 24/7 delivery services	–	–	–
Provision of safe MTP services	–	–	–
Provision of emergency obstetrics care and cesarean services at reachable distance	–	–	–
Child health care strategies			
Specialized care for newborns – facility-based neonatal care	–	–	–
Facility-based integrated management of neonatal and childhood illnesses	–	–	–
Integrated management of childhood illnesses	–	–	–
Home-based neonatal care	–	–	–
Infant and young child feeding	–	–	–
Nutritional rehabilitation centers for malnourished children	–	–	–
Micronutrient (iron and folic acid) supplementation	–	–	–
JSSK	–	–	–
Immunization Increased number of outreach sessions Alternate vaccine delivery vaccinators	–	–	–

**Table 2:** List of outcome variables indicating availability/accessibility of health services, maternal and child health status, and inequalities

Availability/accessibility of services	Maternal health	Child health	Maternal and child health inequalities
Average distance (km) to health facilities Average distance (km) at which doctor/specialist (pediatrician, obstetrician/gynecologist) is available Average distance (km) at which basic and essential diagnostics are available Average distance (km) at which facility for hospitalization for severe illnesses or complications is available Availability of infrastructure like health center building Availability of facilities within the health centers Availability of staff	Impact indicators Maternal mortality ratio Total Fertility Rate Marriage and fertility Percentage of girls marrying before the age of 18 Percentage of births of order 3 and above Sex ratio at birth Percentage of women aged 20–24 reporting birth of order 2 and above. Percentage of births to women aged 15–19, out of total births Marriage and fertility Mothers registered in the first trimester when they were pregnant with last live birth/still birth (%) Mothers who had at least 3 antenatal care visits during their last pregnancy (%) Mothers who got at least one TT injection when they were pregnant with their last live birth/still birth (%) # Institutional births (%) Delivery at home assisted by a doctor/nurse/LHV/ANM (%) Mothers who received postnatal care within 48 hours of delivery of their last child (%) Family planning, current use Any method (%) Any modern method (%) Female sterilization (%) Male sterilization (%) IUD (%) Pill (%) Condom (%) Unmet need for family planning: Total unmet need (%)	Impact indicators Under-5 mortality rate Infant mortality rate Neonatal mortality rate Immunization status: Children (aged 12–23 months) fully immunized (BCG, 3 doses each of DPT and polio, and one dose of measles vaccine) (%) Children (aged 12–23 months) who have received BCG (%) Children (aged 12–23 months) who have received 3 doses of polio vaccine (%) Children (aged 12–23 months) who have received 3 doses of DPT vaccine (%) Children (aged 12–23 months) who have received measles vaccine (%)	Geographical inequalities Urban–rural differences/ratios in maternal and child health Socioeconomic inequalities Socioeconomic differences/ratios in maternal and child health Sex inequalities Female and male child health differences/ratios

#### Quantitative data analysis

The implementation status of the NRHM's health sector plans will be categorized into fully implemented, partially implemented, or not at all implemented, depending upon the utilization of the budget sanctioned for implementation of that plan at the end of 2012 (Table 1). The implementation status of the overall NRHM plan will be based upon the status of individual health sector plans. If all the plans have been fully implemented, the overall NRHM plans will also be considered fully implemented, if partially then partial, if not implemented at all then not at all. Health sector plans will also be differentiated according to whether these resulted in desired action or not, so as to find out which policy measures are effective. The maternal and child health indicators will be compared before, during, and after the introduction of the NRHM, from 2002 to 2012, to assess improvements in maternal and child health outcomes in Haryana. Because the NRHM is implemented in all areas in Haryana, the situation during the pre-NRHM implementation period will serve as a control. Impact indicators such as mortality rates (maternal mortality ratio and child mortality rates) will be compared at the state level for Haryana. The main variables to be compared between 2005 and 2012 are shown in Tables 1 and 2.

Geographical, socioeconomic, and sex inequality in maternal and child health will be assessed by estimating the relative and absolute differences (range) in maternal and child health indicators between urban and rural areas, between the most advantaged and least advantaged socioeconomic groups (excluding maternal and child mortality indicators), and between male and female children. Overall rates and inequalities expressed in terms of ratios and rate differences will be compared across the relevant time period before, during, and after the NRHM. The *p*-value will be considered significant at 95% confidence intervals. Data will be analyzed using Excel and SPSS version 16. Using predesigned methods to extract data from the available documents will minimize bias. In the time period covered (including the introduction of the NRHM), inequalities in child and maternal health indicators may have decreased, but time-dependent changes (other than the introduction of the NRHM) may have occurred simultaneously (e.g. decreased income inequality, increased gross domestic product, other policies/regulations). Hence, potential confounders include sociodemographic variables such as wealth index and education status. Because we only have a pre-versus-post comparison, ‘trends’ of possible confounders will also be identified. Information pertaining to confounding variables will be extracted from demographic health surveys, and multivariate logistic regression analyses will be done.

The findings of the QUAN and QUAL parts of study will be combined during the interpretation stage, using the QUAL data to explain the results of the QUAN study.

### Qualitative study

#### Extent of implementation and effectiveness of NRHM plans

The perceptions of program managers, service providers, community, and target group will be explored regarding the extent of implementation of NRHM plans, the affordability and accessibility of healthcare services, and the extent of improvement in geographical, socioeconomic, and sex differences in maternal and child health outcomes during the year 2013–2014. The study sample used for the interviews and focus groups will include the Mission Director of NRHM, Haryana; Program Managers (State/District Maternal and Child Health Officers or NRHM Nodal Officers); community/opinion leaders including religious leaders, group leaders, village heads or priests, and so on; mothers with children aged under 5; and service providers including medical officers, ANMs, and ASHAs.

The sampling method will be purposive. One CHC, one PHC, one subcenter, one village from a rural area, and a city/town and a slum from an urban area will be selected from the districts of Ambala and Mewat. As there is variability within the districts regarding maternal and child health status, with certain blocks performing better than others, we will select well-performing and poorly performing health centers within the districts of Ambala and Mewat. FGDs will be held with the service providers at each level, that is, ASHAs at the village level, ANMs at the subcenter level, medical officers at the PHC level, and senior medical officers at the CHC level. Each FGD will include 5–10 participants. In-depth interviews will be conducted with the Mission Director of NRHM of the state, the program managers of maternal and child health at the state and district level, the community leaders, and at least two mothers at each level (district, CHC, PHC, subcenter/village). FGDs and in-depth interviews will be conducted until data saturation is achieved.

The approach to be used in the qualitative study will be based on grounded theory. Guides for the in-depth interviews and FGDs will be prepared and used after pretesting in the community (Supplementary files). All the FGDs and in-depth interviews will be audio- and video-recorded after obtaining written informed consent, so that verbal and non-verbal responses can be recorded. Manual recording will also be done as a backup.

#### Qualitative data analysis

All the information obtained from the FGDs and in-depth interviews will be first transcribed in Hindi/local language using the audio and video recordings and the manual recordings. The transcribed version will then be translated into English. Memos will be assigned and two independent coders (authors) who are trained in qualitative analysis will identify codes. Thematic analysis of the content will then be done either manually or using Nvivo statistical software to identify the patterns.

## Recommendations

Based upon the evidence obtained through this mixed-methods study, appropriate recommendations will be made for policy makers and program managers regarding implementing strategies for maternal and child healthcare and to reduce the health inequalities in Haryana as well as India as a whole.

## Ethics and dissemination

Ethical approval has been obtained from the Post Graduate Institute of Medical Education and Research (PGIMER) Ethics Committee. A study information sheet will be provided and written informed consent will be obtained from participants of the FGDs and in-depth interviews.

## Discussion

The results of this study will provide important information on the trends in the implementation of NRHM's health sector plans and its effectiveness in improving maternal and child health outcomes and reducing geographic, socioeconomic, and sex inequalities in maternal and child health. The evidence will be collected through a novel and rigorous mixed-methods study conducted in Haryana, so that the findings can guide the effective implementation of NRHM plans to reduce these inequalities in maternal and child health and achieve the intended maternal and child health goals. We chose a mixed-methods approach because the use of quantitative and quantitative methods will enable us to not only estimate the extent of implementation and effectiveness of the multiple-strategy intervention in reducing maternal and child health inequalities in Haryana (QUAN study findings) but also explain the possible causes of these results (from the QUAL study findings). The partially mixed sequential equal status design will afford us the flexibility to use existing quantitative data from demographic surveys held before, during, and after the NRHM implementation [DLHS 2 (2004–2005), DLHS 3 (2007–2008), and DLHS 4 (2012–2013)] for trend analysis. The qualitative study will be performed last so as to ascertain the status of implementation of NRHM at its flag end (26). The aim of this approach is to ascertain the status of implementation of NRHM plans objectively as per the amount of budget spent on various health sector plans, as well as from the perspective of providers, managers, and the community; and to assess the status of maternal and child health indicators and inequalities across geographical, socioeconomic, and sex domains after the implementation of the NRHM.

Most of the previous studies on maternal and child health inequalities in India have used the data of demographic health surveys collected prior to the implementation of the NRHM, so we do not know the present status of these inequalities after the targeted intervention (NRHM). Pathak and colleagues reported that, despite several governmental efforts to increase access and coverage of delivery services to the poor, the poor did not use skilled birth attendance (SBA), and even if they used SBA, they were more likely to use the private providers. These authors, however, provided no suggestions to increase public sector facilities utilization (24). Goli et al. recommended adopting different health policy interventions, in accordance with the pattern of varying contributions of socioeconomic factors to child health inequalities, between more developed southern Indian states and less developed states (33). Pallikadavath and colleagues performed a quantitative assessment of maternal and child health inequalities ‘within’ and ‘between’ states. They emphasized that a policy and programming was needed to address ‘within-state’ inequalities as a priority (aimed at ensuring the availability of all-weather roads and primary schools), because these, more than ‘between-state’ inequalities, affected maternal and child health inequalities by influencing the availability and accessibility of these services (25). All these studies suggest that state-specific actions are necessary to deal with inequalities.

The planning commission's evaluation of the NRHM during its implementation does not include Haryana, and did not measure maternal and child health inequalities. The advantage of the current study is that it will use an approach for program assessment that is more holistic in nature, and will be helpful in identifying the barriers and facilitators for implementing the strategies, as compared to one-time assessments, which are generally cross-sectional in nature, and provide no explanations for the findings. Recommendations based upon holistic program assessments are likely to deliver a more complete and complex picture, and hence to be more relevant for policy making. In addition, by qualitatively comparing the implementation status in well-performing and poorly performing districts, issues and bottlenecks in poorly performing districts can be brought to the attention of the policy makers, so that immediate action can be taken to overcome these barriers. The results of our study will not only benefit Haryana but might also help the whole nation to improve the planning and implementation of services aimed at improving maternal and child health and reducing the major geographical, socioeconomic, and sex-related inequalities in health in India.

## Supplementary Material

Effectiveness of a multiple-strategy community intervention to reduce maternal and child health inequalities in Haryana, North India: a mixed-methods study protocol
