# Supplementary material for: Effectiveness of a multiple-strategy community intervention to reduce maternal and child health inequalities in Haryana, North India: a mixed-methods study protocol
Source: Glob Health Action. 2015 Feb 10;8:10.3402/gha.v8.25987. doi: 10.3402/gha.v8.25987 (PMC4326669; doi:10.3402/gha.v8.25987)
Supplement: Effectiveness of a multiple-strategy community intervention to reduce maternal and child health inequalities in Haryana, North India: a mixed-methods study protocol [file GHA-8-25987-s001.docx]

**Received:** 2014-09-11, Revised: 2015-01-13, Accepted:  2015-01-16, Published: xx

Responsible editor: Stig Wall, Umeå University, Sweden

**Title**

Effectiveness of a multiple-strategy community intervention to reduce maternal and child health inequalities in Haryana, North India: A mixed-methods study protocol.

**Short Running Head**

Community intervention to reduce maternal and child health inequalities

**Name of Authors**

**Madhu Gupta**

Associate Professor, School of Public Health, PGIMER, Chandigarh, India

MG: madhugupta21@gmail.com

**Federica Angeli**

Assistant Professor of Health Care Management, Department of Health Services Research, CAPHRI, Maastricht University, Maastricht, The Netherlands

FA: federica.angeli@maastrichtuniversity.nl

**Onno C.P. van Schayck**

Scientific Director of CARE and Professor of Preventive Medicine, Department of Family Practice, CAPHRI, Maastricht University, Maastricht, The Netherlands

OvS: onno.vanschayck@maastrichtuniversity.nl

**Hans Bosma**

Professor of Social Epidemiology, Department of Social Medicine, CAPHRI, Maastricht University, Maastricht, The Netherlands

HB: hans.bosma@maastrichtuniversity.nl

**Word count**

Abstract: 300, Text: 4913, references: 33, Tables:2, Figures:1, Appendices: 2

**Corresponding Author**

Dr Madhu Gupta, Associate Professor, School of Public Health, Room No. 130, PGIMER, Chandigarh, India

Mobile: +919914208226

Phone: +911722690727

Email:madhugupta21@gmail.com

**Funding**

This work was supported by National Rural Health Mission, Haryana, India**.**

**Acknowledgements**

We would like to acknowledge the support provided by Dr Rakesh Gupta, IAS, PhD (JHU), Mission Director, National Rural Health Mission, Government of Haryana, India, for sanctioning the funds for this study. I am thankful to Professor Rajesh Kumar, Head of School of Public Health and Dr Manmeet Kaur, Associate Professor, School of Public Health, PGIMER, Chandigarh, for their help in planning the thesis. I am grateful to Dr Shankar Prinja, Assistant Professor of Health Economics, School of Public Health, PGIMER, Chandigarh for sharing the data of the concurrent evaluation of NRHM, Haryana for the year 2012-13.

**Authors’ contributions**

**MG**

1) substantial contributions to conception and design, acquisition of data, analysis and interpretation of data

2) involved in drafting the manuscript

3) given final approval for the version to be published

**FA**

1) substantial contributions to conception and design, acquisition of data, analysis and interpretation of data

2) involved in drafting the manuscript and revising it critically for important intellectual content

3) given final approval for the version to be published

**OVS**

1) substantial contributions to conception and design, and interpretation of data

2) involved in drafting the manuscript and revising it critically for important intellectual content

3) given final approval for the version to be published

**HB**

1) substantial contributions to conception and design, data analysis and interpretation of data;

2) involved in drafting the manuscript and revising it critically for important intellectual content

3) given final approval for the version to be published

**Funding Statement**

This study was funded by National Rural Health Mission, Haryana, India.

**Competing interest**

There are no competing interests, as we, as an independent agency, will objectively evaluate NRHM policy measures.

**Abstract**

**Background**

A multiple-strategy community intervention, known as National Rural Health Mission (NRHM), launched in India to improve the availability of and access to better-quality healthcare, especially for rural, poor mothers and children. The final goal of the intervention is to reduce maternal and child health inequalities across geographical areas, socioeconomic status groups, and gender of child. Extensive, in-depth research is necessary to assess the effectiveness of NRHM, on multiple outcome dimensions. This paper presents the design of a new study, able to overcome the shortcomings of previous research.

**Objective**

To propose a comprehensive, methodologically sound protocol to assess the extent of implementation and the effectiveness of NRHM measures to improve maternal and child health outcomes and reduce maternal and child health inequalities.

**Design**

A mixed-methods approach (quantitative and qualitative) is proposed for this study in Haryana, a North Indian State. NRHM’s health sector plans included health system strengthening, specific maternal and child healthcare strategies, and communitization. Mission documents and reports on progress, financial monitoring, common and joint review will be reviewed in-depth to assess the extent of the implementation of plans. Data on maternal and child health indicators will be obtained from demographic health surveys held before, during, and after the implementation of the first phase of the NRHM (2005 to 2012) and compared over time. Differences in maternal and child health indicators will be used to measure maternal and child health inequalities; these will be compared pre- and post-NRHM. Focus group discussions with service providers and in-depth interviews with program managers, community representatives, and mothers will be conducted until data saturation is achieved, in two districts of Haryana. Using Nvivo software, an inductive qualitative content analysis will be performed to search for the broader themes across the interviews and focus group discussions. Ethical approval was obtained from the Ethics Committee of the Post Graduate Institute of Medical Education and Research.

**Keywords**

National Rural Health Mission, India, health inequalities, mixed-methods approach, maternal health, child health, health indicators

**Main Text**

**Introduction**

Achieving millennium development goals 4 and 5 – i.e., reducing under-five child mortality by two thirds and maternal mortality by three quarters between 1990 and 2015 – is among the highest priorities on India’s national health agenda [^[[1]](#endnote-1)^]. Between 2005 and 2012, India’s total spending on health increased from 0.9% of gross domestic product to nearly 2%, but maternal and child health indicators have not improved correspondingly [^[[2]](#endnote-2)^].

*Problem statement*

The maternal mortality rate (MMR) is still as high as 178 maternal deaths per hundred thousand live births [^[[3]](#endnote-3)^] and the infant mortality rate (IMR) is 42 infant deaths per thousand live births [^[[4]](#endnote-4)^]. There is geographical inequality in maternal and child health outcomes. For example, IMR is higher in rural as compared to urban areas (48 against 28 deaths per thousand live births) [4]. Large geographical and socioeconomic inequalities in maternal and child health status and access to health services continue to persist in India and have even widened across states, between rural and urban areas, and within communities [^[[5]](#endnote-5)^]. Singh et al reported inequality regarding advice during the antenatal period and the coverage of essential postnatal care, which is provided disproportionately more frequently among the rich [^[[6]](#endnote-6)^, ^[[7]](#endnote-7)^]. Nayar reported maternal and child health inequalities across different caste groups [scheduled castes (SC), scheduled tribes (ST) and other backward castes (OBC) and general castes] in India. SC/ST/OBC (lower castes) represent communities belonging to lower socioeconomic groups with a poor maternal and child health status as compared to higher castes [^[[8]](#endnote-8)^]. Pathak et al reported a disproportionately concentrated malnutrition burden among poor children and slow changes in child malnutrition in India during 1992-2006, coupled with a concomitant rise in economic inequalities [^[[9]](#endnote-9)^]. Pradhan et al reported that poor household economic status (46%), mother's illiteracy (35%) and rural residence (15%) explained 96% of the total socioeconomic inequalities in child survival at the national level [^[[10]](#endnote-10)^]. Gender inequality among children is another area of concern, especially among North Indian states, involving a strong preference for sons, female feticide, and declining sex ratio at birth [^[[11]](#endnote-11)^]. Gender disparity in immunization programs favoring males has been reported in urban areas, developed states and Muslim communities in India [^[[12]](#endnote-12)^]. Better healthcare-seeking behaviors of caregivers for sick male children as compared to sick female children further add to the gender-related health inequalities.

This persistence of maternal and child health inequalities highlights the need to assess how the existing national health programs or policies on maternal and child health are being implemented. Simultaneously, it indicates the need for studies on the effectiveness of these programs, as these are highly resource-intensive. Such assessments can inform policy makers in resource-constrained countries like India on ways to improve the policy or implementation strategy of these interventions.

*Study population*

Studying maternal and child health inequalities in the Indian state of Haryana is worthwhile as it is representative of other North Indian states with similar socioeconomic development and sociocultural factors, such as the preference to have sons, female feticide, lower sex ratios and lower social status of women. At the same time, Haryana represents a unique context by being a prosperous state with a rising economy but with unequal distribution of resources, which has led to wide intra-state and inter-district differences in terms of provision of basic infrastructure like water, roads, schools, hospitals etc. Despite being one of the richer states, reporting the highest per capita income in the country at Rs 1,09,064 (USD 1947.6) during 2012-13, maternal and child health indicators are not the best in the country [^[[13]](#endnote-13)^]. Although the maternal mortality rate has declined from 176 (for the year 1999-2001) [^[[14]](#endnote-14)^] to 146 deaths per one hundred thousand live births (for the year 2010-12) [3], it still lags behind the goal of reducing it to below 100 by 2015 [1]. There are marked geographical differences in maternal and child health: the infant mortality rate is higher in rural areas (46 per thousand live births) compared to urban areas (33 per thousand live births) [4]. The child sex ratio at birth declined from 964 in the 2001 census to 830 per thousand males in the 2011 census [12]. There is a clear problem of female feticide and poor health-seeking behavior for daughters. [^[[15]](#endnote-15)^, ^[[16]](#endnote-16)^, ^[[17]](#endnote-17)^, ^[[18]](#endnote-18)^]. All this provides us with an excellent opportunity to study inequalities in this state.

*Current and past interventions/current state, new interventions*

Past interventions to improve maternal and child health were initially implemented as vertical programs, like the Family Welfare Program (1952), Acute Diarrheal Disease Control Program (1978), Acute Respiratory Infections Control Program (1978) and Universal Immunization Program (1985). These initiatives were later merged, initially as the Safe Childhood and Safe Motherhood Program (CSSM, 1992) and then as the Reproductive and Child Health Program (RCH I, 1997-2005), as it was realized that improving maternal health is imperative to improving child health [^[[19]](#endnote-19)^]. However the main objective in these earlier programs was to improve the maternal and child health indicators and increase their survival, and not much emphasis was put on reducing inequalities. Realizing this gap in implementation, a national multiple-strategy community intervention was launched, known as the National Rural Health Mission (NRHM), by the Ministry of Health and Family Welfare of the Government of India [started during 2005 in the 11^th^ health plan (2005 to 2012), and continued in 12^th^ health plan (2012 to 2017)] with the aim to reduce health inequalities by improving the availability of and access to better-quality healthcare, especially for people residing in rural areas (to reduce geographical inequality), for the poor (to reduce socioeconomic inequality), and for women and children (to reduce gender inequality) [^[[20]](#endnote-20)^]. NRHM’s health sector plans included health system strengthening, specific maternal and child healthcare strategies/schemes (RCH-II), and communitization (delegating powers to and empowering the community to monitor the healthcare delivery system) [^[[21]](#endnote-21)^]. Details of these plans are given in Appendix 1. Briefly, health system strengthening included making available mobile medical units (MMUs) and patient transport services, strengthening the health infrastructure, providing free drugs and logistics, and providing telemedicine facilities. Maternal and child health schemes included cash incentives for hospital deliveries, free delivery services for pregnant women and treatment of neonatal illnesses in hospitals, reimbursements of travel cost to hospitals and appointing Accredited Social Health Activists (ASHAs) to promote the access to improved healthcare at household level in villages. The intention was to reduce the infant mortality rate to 30/1,000 live births, maternal mortality to 1/1,000 live births, and the total fertility rate to 2.1 by 2012. It was further realized that NRHM strategies were not covering the urban poor, whose condition was even worse than that of the rural population. Hence, NRHM was renamed National Health Mission (2012), and now covers the slum population as well.

*Previous assessments and their strengths/weaknesses*

The planning commission of India had the NRHM schemes evaluated in seven states (Uttar Pradesh, Madhya Pradesh, Jharkhand, Orissa, Assam, Jammu and Kashmir and Tamil Nadu) during the fourth year of its implementation (2009-10) and assessed the availability, adequacy and utilization of maternal and child health services [^[[22]](#endnote-22)^]. They conducted cross-sectional surveys, focus group discussions and in-depth interviews with stakeholders. They observed some improvements in the availability and utilization of maternal and child health services in rural areas, and recommended further strengthening of health facilities. The strength of their study lies in the inclusion of a qualitative assessment of the program that gave insight into the implementation process. However, their evaluation was limited by the lack of assessment of the extent of implementation of NRHM schemes, including budget sanctioned and spent on NRHM schemes, the lack of comparison of results with the situation before the implementation of the NRHM, the lack of measurement of maternal and child health inequalities, and the lack of interpretation of quantitative data and qualitative data by a mixed-methods approach. Since NRHM had two more years to go at the time of the planning commission’s evaluation, it represented a mid-term evaluation. The present study intends to overcome the above limitations by assessing the extent of implementation of NRHM schemes in the maternal and child health care sector, including the budgetary outlays for maternal and child health schemes through the NRHM period (2005-12) and its effectiveness, by comparing the situation before, during and after the implementation of the NRHM using a mixed-methods approach. In another study by Mukherjee et al, 100 rural doctors from the states of Orissa, Assam, Jharkhand and Chattisgarh were interviewed to analyze the effectiveness of the NRHM in improving the availability and accessibility of health services in rural areas. They concluded that it was not 100% effective and there were inefficiencies in terms of infrastructure and manpower [^[[23]](#endnote-23)^]. Earlier surveys did report on the effectiveness of health services, but none reported on maternal and child health inequalities. Also, no previous study has been conducted in Haryana state. State-specific information is necessary, as each state is different, having its own unique cultural, social, and demographic backgrounds and problems. Since the causes of maternal and child health inequalities vary across states, solutions to bridge the gaps thus have to be tailor-made [^[[24]](#endnote-24)^,^[[25]](#endnote-25)^].

It is against this background that the present mixed-methods study was designed, to quantify the extent of implementation of NRHM’s maternal and child health-related plans in the healthcare sector, to quantify NRHM’s effectiveness in terms of reducing geographical, socioeconomic, and gender inequalities and improving the overall maternal and child health outcomes, as well as to qualitatively ascertain the extent to which maternal and child health strategies in the NRHM were implemented and were effective in tackling the inequalities and outcomes, and to formulate evidence-based recommendations for bridging the health inequalities in Haryana state.

**Study Design**

A mixed-methods approach will be used in this study, involving a partially mixed sequential equal status design in terms of the Leech classification [^[[26]](#endnote-26)^]. Partially mixed design implies that mixing of qualitative (QUAL) and quantitative (QUAN) data will be done at interpretation level (i.e. the quantitative data will be linked to and explained by qualitative results); sequential means that the qualitative data will be collected after the quantitative data collection; and equal status denotes that both qualitative and quantitative data will be given the same importance at the time of interpretation. The quantitative part of the mixed-method study will reveal the trends and differences in rates for maternal and child health outcomes and inequalities before, during, and after NRHM implementation, whereas the qualitative study will provide explanations for these findings, which will be used to formulate evidence-based recommendations for implementing the program in a more effective way, so as to achieve the intended maternal and child health goals.

**Setting**

This study will be done in Haryana state in India. A state is divided into many administrative districts, which include several administrative blocks. The Chief Medical Officer (CMO) is the overall person in charge of implementing national health programs at the district level. There are several program officers, one for each program, who report to the CMO. There is a three-tier system of health care infrastructure in each district: at grass-root level, there is a sub-center catering for a population of 5,000, a primary health center (PHC) catering for a population of 30,000, and a community health center (CHC) catering for a population of 100,000. Above the CHC (block) level, there is either a sub-district or district hospital. A doctor is available at the PHC level and above. At the sub-health center, an Auxiliary Nurse Midwife (ANM) is responsible for implementing maternal and child health programs. She is assisted by an Accredited Social Health Activist (ASHA) [6] and a child care volunteer called Anganwadi Worker (AWW) for each village or population of 1,000 [9]. Community groups include Panchayati Raj Institutions, village health committees, and self-help groups in villages [^[[27]](#endnote-27)^,^[[28]](#endnote-28)^].

Haryana has 21 districts, and has a population of 25,353,081 (70% rural), a birth rate of 21.6 and a mortality rate of 6.4 deaths per thousand mid-year population [4 , 10]. For the qualitative study, we will select a well performing (Ambala) and a poorly performing district (Mewat) of Haryana. This selection will allow us to obtain a better contextual understanding of two extreme situations and to learn which scheme works better in a particular situation, by exploring the perceptions and beliefs of service providers, community representatives, and mothers regarding the implementation status and effectiveness of NRHM's maternal and child health schemes. (Figure 1). Criteria for labeling the district as well or less well performing are based on the District Level Household Survey 3 (DLHS-3, 2007-08) [^[[29]](#endnote-29)^]. Maternal and child health indicators for comparison included age at marriage below 18 years (3% in Ambala v/s 43% in Mewat), teenage pregnancies (0.9% in Ambala v/s 9.3% in Mewat), availability of antenatal care (83% in Ambala v/s in 53% Mewat), institutional births (55% in Ambala v/s 15% in Mewat), availability of postnatal care (70% v/s 34%), and fully immunized children (92% Ambala v/s 20% Mewat). About 51% of the population of Ambala have a high standard of living index, compared to 11% in Mewat. Although differing in the above characteristics, the Ambala and Mewat districts have a similar population size (Ambala 1,128,350; Mewat 1,089,263) and density (Ambala 717; Mewat 723 per sq km).

**Data Collection and Analyses**

Quantitative study

*Extent of implementation of NRHM plans*

Information will be obtained via an in-depth review of the NRHM mission document [21], the progress reports of the NRHM, approved state program implementation plans, and financial monitoring reports (FMRs), in order to obtain information on budgets approved and spent for each activity planned in the financial years 2005 to 2012. Independent evaluation reports, such as those by the Common Review Mission (CRM) [^[[30]](#endnote-30)^] and Joint Review Mission (JRM) [^[[31]](#endnote-31)^] will also be reviewed. CRM reports are an important component of the overall monitoring and evaluation framework envisaged in the NRHM implementation framework. The CRM undertakes spot appraisal of the health system and reflects on the success of the strategies and policies with the aim of identifying the need for potential mid-course corrections in implementation. Seven such reviews have been conducted so far,s and Haryana state was covered in the 3^rd^, 5^th^ and 7^th^ CRMs. Data pertaining to maternal and child health indicators in these documents will be recorded on a predesigned form to avoid selection bias. This will prevent rejection or acceptance of “bad” data on arbitrary grounds instead of according to previously stated criteria as listed in predesigned form.

*Effectiveness of NRHM plans*

Information on the status of maternal and child health indicators will be obtained from the National Family Health Survey (2005-06) [^[[32]](#endnote-32)^], the District Level Household Survey [DLHS 2 (2002-03), the DLHS 3 (2007-08), and the DLHS 4 (2012-13)] [29 ]. DLHS 2 represents the situation before, DLHS 3 that during and DLHS 4 that after NRHM implementation. These surveys provide consistent and reliable estimates of fertility, mortality, family planning, utilization of maternal and child healthcare services, and other related indicators at both the national and state levels. Maternal and infant mortality rates at the Haryana state level will be obtained from the Sample Registration System [3,4].

*Variables*

Implementation variables include NRHM health sector measures (Table 1), while socio-demographic variables include wealth index, education, caste, and religion, as available from demographic surveys. Outcome variables for this study are listed in Table 2. These include maternal and child health indicators, maternal and child health inequalities across the socioeconomic, geographical, and gender gradients and indicators on the access/availability of maternal and child health services.

*Quantitative Data Analysis*

The implementation status of the NRHM’s health sector plans will be categorized into fully implemented, partially implemented, or not at all implemented, depending upon the utilization of the budget sanctioned for implementation of that plan at the end of 2012. (Table 1). The implementation status of the overall NRHM plan will be based upon the status of individual health sector plans. If all the plans have been fully implemented, the overall NRHM plans will also be considered fully implemented, if partially then partial, if not implemented at all then not at all. Health sector plans will also be differentiated according to whether these resulted in desired action or not, so as to find out which policy measures are effective. The maternal and child health indicators will be compared before, during and after the introduction of the NRHM, from 2002 to 2012, to assess improvements in maternal and child health outcomes in Haryana. Since the NRHM is implemented in all areas in Haryana, the situation during the pre-NRHM implementation period will serve as a control. Impact indicators like mortality rates (maternal mortality ratio and child mortality rates) will be compared at state level for Haryana. The main variables to be compared between 2005 and 2012 are shown in Tables 1 and 2.

Geographical, socioeconomic, and gender inequality in maternal and child health will be assessed by estimating the relative and absolute differences (range) in maternal and child health indicators between urban and rural areas, between the most advantaged and least advantaged socioeconomic groups (excluding maternal and child mortality indicators), and between male and female children. Overall rates and inequalities expressed in terms of ratios and rate differences will be compared across the relevant time period before, during, and after the NRHM. The P-value will be considered significant at 95% confidence intervals. Data will be analyzed using Excel and SPSS version 16. Using predesigned methods to extract data from the available documents will minimize bias. In the time period covered (including the introduction of the NRHM), inequalities in child and maternal health indicators may have decreased, but time-dependent changes (other than the introduction of the NRHM) may have occurred simultaneously (e.g. decreased income inequality, increased gross domestic product, other policies/regulations). Hence, potential confounders include socio-demographic variables like wealth index and education status. Since we only have a pre-versus-post comparison, ‘trends’ of possible confounders will also be identified. Information pertaining to confounding variables will be extracted from demographic health surveys, and multivariate logistic regression analyses will be done.

The findings of the QUAN and QUAL parts of study will be combined during the interpretation stage, using the QUAL data to explain the results of the QUAN study.

Qualitative study

*Extent of implementation and effectiveness of NRHM plans*

The perceptions of program managers, service providers, community, and target group will be explored regarding the extent of implementation of NRHM plans, the affordability and accessibility of healthcare services, and the extent of improvement in geographical, socioeconomic, and gender differences in maternal and child health outcomes during the year 2013-14. The study sample used for the interviews and focus groups will include the Mission Director of NRHM, Haryana; Program Managers (State/District Maternal and Child Health Officers or NRHM Nodal Officers); community/opinion leaders including religious leaders, group leaders, village heads or priests etc.; mothers with children aged under five; and service providers including Senior/Medical officers, Auxiliary Nurse Midwives (ANMs), and Accredited Social Health Activists (ASHAs).

The sampling method will be purposive. One CHC, one PHC, one sub-center, one village from a rural area, and a city/town and a slum from an urban area will be selected from the Ambala and Mewat districts. As there is variability within the districts regarding maternal and child health status, with certain blocks performing better than others, we will select well-performing and poorly performing health centers within the Ambala and Mewat districts. Focus group discussions (FGDs) will be held with the service providers at each level, i.e., ASHAs at the village level, ANMs at the sub-center level, medical officers at the PHC level and senior medical officers at the CHC level. Each FGD will include 5-10 participants. In-depth interviews will be conducted with the Mission Director of NRHM of the state, the program managers of maternal and child health at the state and district level, the community leaders, and at least two mothers at each level (district, CHC, PHC, sub-center/village). FGDs and in-depth interviews will be conducted until data saturation is achieved.

The approach to be used in the qualitative study will be based on grounded theory. Guides for the in-depth interviews and FGDs will be prepared and used after pretesting in the community (Appendix 2). All the FGDs and in-depth interviews will be audio- and video-recorded after obtaining written informed consent, so that verbal and non-verbal responses can be recorded. Manual recording will also be done as a backup.

*Qualitative Data Analysis*

All the information obtained from the FGDs and in-depth interviews will be first transcribed in Hindi/local language using the audio and video recordings and the manual recordings. The transcribed version will then be translated into English. Memos will be assigned and two independent coders (authors) who are trained in qualitative analysis will identify codes. Thematic analysis of the content will then be done either manually or using Nvivo statistical software to identify the patterns.

**Recommendations**

Based upon the evidence obtained through this mixed-methods study, appropriate recommendations will be made for policy makers and program managers regarding implementing strategies for maternal and child healthcare and to reduce the health inequalities in Haryana as well as India as a whole.

**Ethics and dissemination**

Ethical approval has been obtained from the Post Graduate Institute of Medical Education and Research (PGIMER) Ethics Committee. A study information sheet will be provided and written informed consent will be obtained from participants of the focus group discussions and in-depth interviews.

**Discussion**

The results of this study will provide important information on the trends in the implementation of NRHM’s health sector plans and its effectiveness in improving maternal and child health outcomes and reducing geographic, socioeconomic, and gender inequalities in maternal and child health. The evidence will be collected through a novel and rigorous mixed-methods study conducted in Haryana, so that the findings can guide the effective implementation of NRHM plans to reduce these inequalities in maternal and child health and achieve the intended maternal and child health goals. We chose a mixed-methods approach as the use of quantitative as well as quantitative methods will enable us to not only estimate the extent of implementation and effectiveness of the multiple strategy intervention in reducing maternal and child health inequalities in Haryana (QUAN study findings) but also explain the possible causes of these results (from the QUAL study findings). The partially mixed sequential equal status design will afford us the flexibility to use existing quantitative data from demographic surveys held before, during, and after the NRHM implementation [DLHS 2 (2004-05), DLHS 3 (2007-08), DLHS 4 (2012-13)] for trend analysis. The qualitative study will be performed last, so as to ascertain the status of implementation of NRHM at its flag end [26]. The aim of this approach is to ascertain the status of implementation of NRHM plans objectively as per the amount of budget spent on various health sector plans, as well as from the perspective of providers, managers, and the community; and to assess the status of maternal and child health indicators and inequalities across geographical, socioeconomic, and gender domains after the implementation of the NRHM.

Most of the previous studies on maternal and child health inequalities in India have used the data of demographic health surveys collected prior to the implementation of the NRHM, so we do not know the present status of these inequalities after the targeted intervention (NRHM). Pathak and colleagues reported that, despite several governmental efforts to increase access and coverage of delivery services to the poor, the poor did not use skilled birth attendance (SBA), and even if they used SBA, they were more likely to use the private providers. These authors, however, provided no suggestions to increase public sector facilities utilization [24]. Goli et al recommended adopting different health policy interventions, in accordance with the pattern of varying contributions of socioeconomic factors to child health inequalities, between more developed southern Indian states and less developed states [^[[33]](#endnote-33)^]. Pallikadavath and colleagues performed a quantitative assessment of maternal and child health inequalities ‘within’ and ‘between’ states. They emphasized that a policy and programming was needed to address 'within-state' inequalities as a priority (aimed at ensuring the availability of all-weather roads and primary schools), as these, more than ‘between-state’ inequalities affected maternal and child health inequalities by influencing the availability and accessibility of these services [25]. All these studies suggest that state specific actions are necessary to deal with inequalities.

The planning commission’s evaluation of the NRHM during its implementation does not include Haryana state, and did not measure maternal and child health inequalities. The advantage of the current study is that it will use an approach for program assessment that is more holistic in nature, and will be helpful in identifying the barriers and facilitators for implementing the strategies, as compared to one-time assessments, which are generally cross-sectional in nature, and provide no explanations for the findings. Recommendations based upon holistic program assessments are likely to deliver a more complete and complex picture, and hence to be more relevant for policy making. Additionally, by qualitatively comparing the implementation status in well performing and poorly performing districts, issues and bottlenecks in poorly performing districts can be brought to the attention of the policy makers, so that immediate action can be taken to overcome these barriers. The results of our study will not only benefit Haryana, but might also help the whole nation to improve the planning and implementation of services aimed at improving maternal and child health and reducing the major geographical, socioeconomic, and gender-related inequalities in health in India.

**Figures**

**Figure 1. Map of Haryana showing districts selected for qualitative study, 2013**


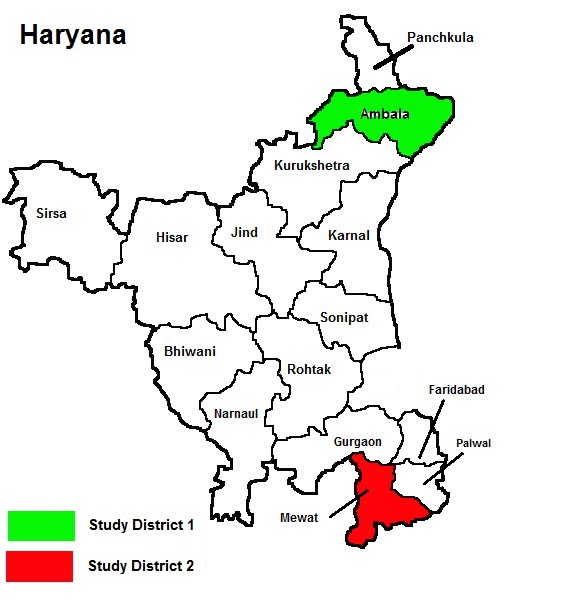


**Table 1. Implementation status of NRHM health sector plans.**

| **NRHM Plans**  **(Independent variables)** | **Implementation status** | | | |
| --- | --- | --- | --- | --- |
|  | **Full** | | **Partial** | **None** |
| **1. Health System Strengthening** | | | | |
| Mobile medical units with access to hard-to-reach areas | | **-** | **-** | **-** |
| Patient transport service/referral services | | **-** | **-** | **-** |
| Infrastructure development and strengthening: construction of new healthcare facilities for universal access to primary healthcare as per the norms, and strengthening of existing facilities as per Indian Public Health Standards (IPHS) | | **-** | **-** | **-** |
| Human resources: availability of additional nurses, doctors, specialists, ANMs, administrative staff on a contractual basis | | **-** | **-** | **-** |
| Drugs and logistics (free essential medicines at all healthcare facilities) | | **-** | **-** | **-** |
| Telemedecine | | **-** | **-** | **-** |
| **2. Communitization** | | | | |
| Accredited Social Health Activist (ASHA) | | **-** | **-** | **-** |
| Village health and sanitation committees | | **-** | **-** | **-** |
| Village health and nutrition days | | **-** | **-** | **-** |
| *Rogi Kalyan Samities* (patient welfare committees in the hospitals with members also deriving from the community) | | **-** | **-** | **-** |
| **3. Maternal Healthcare Strategies** | | | | |
| *JSY- Janani Suraksha Yojna* | | **-** | **-** | **-** |
| *JSSK-Janani Shishu Suraksha Karyakaram* | | **-** | **-** | **-** |
| Increased number of delivery points with provision of 24/7 delivery services | | **-** | **-** | **-** |
| Provision of safe MTP services | | **-** | **-** | **-** |
| Provision of emergency obstetrics care and cesarean services at reachable distance | | **-** | **-** | **-** |
| **4. Child Health Care Strategies** | | | | |
| Specialized care for newborns – facility-based neonatal care | | **-** | **-** | **-** |
| Facility-based integrated management of neonatal and childhood illnesses | | **-** | **-** | **-** |
| Integrated management of childhood illnesses | | **-** | **-** | **-** |
| Home-based neonatal care | | **-** | **-** | **-** |
| Infant and young child feeding | | **-** | **-** | **-** |
| Nutritional rehabilitation centers for malnourished children | | **-** | **-** | **-** |
| Micronutrient (iron and folic acid) supplementation | | **-** | **-** | **-** |
| JSSK | | **-** | **-** | **-** |
| Immunization  Increased number of outreach sessions  Alternate vaccine delivery vaccinators | | **-** | **-** | **-** |

**Table 2. List of outcome variables indicating availability/accessibility of health services, maternal and child health status, and inequalities**

| **Availability/Accessibility of services** | **Maternal Health** | **Child Health** | **Maternal and Child Health Inequalities** |
| --- | --- | --- | --- |
| Average distance (km) to health facilities  Average distance (km) at which doctor/ specialist (pediatrician, obstetrician/gynecologist) is available  Average distance (km) at which basic and essential diagnostics are available  Average distance (km) at which facility for hospitalization for severe illnesses or complications is available  Availability of infrastructure like health center building  Availability of facilities within the health centers, like  Availability of staff | **Impact indicators**   - Maternal mortality ratio - Total Fertility Rate   **Marriage and fertility**   - Percentage of girls marrying before age 18 years - Percentage of births of order 3 and above - Sex Ratio at birth - Percentage of women ages 20-24 years reporting birth of order 2 and above. - Percentage of births to women at ages 15-19, out of total births   **Maternal Health**   - Mothers registered in the first trimester when they were pregnant with last live birth/still birth (%) - Mothers who had at least 3 ante-natal care visits during their last pregnancy (%) - Mothers who got at least one TT injection when they were pregnant with their last live birth / still birth (%) # - Institutional births (%) - Delivery at home assisted by a doctor/nurse /LHV/ANM (%) - Mothers who received postnatal care within 48 hours of delivery of their last child (%)   **Family planning, current use**   - Any method (%) - Any modern method (%) - Female sterilization (%) - Male sterilization (%) - IUD (%) - Pill (%) - Condom (%)   **Unmet Need for Family Planning:**   - Total unmet need (%) | **Impact indicators**   - Under-5 mortality rate - Infant mortality rate - Neonatal mortality rate   **Immunization Status**   - Children (12-23 months) fully immunized (BCG, 3 doses each of DPT and polio and one dose of measles vaccine) (%) - Children (12-23 months) who have received BCG (%) - Children (12-23 months) who have received 3 doses of polio vaccine (%) - Children (12-23 months) who have received 3 doses of DPT vaccine (%) - Children (12-23 months) who have received measles vaccine (%) | **Geographical inequalities**   - Urban-rural differences/ratios in maternal and child health |
|  |  |  | **Socioeconomic inequalities**   - Socioeconomic differences/ratios in maternal and child health   **Gender Inequalities**   - Female and male child health differences/ratios |

**Appendix 1.**

**National Rural Health Mission’s Health Sector Plans**

**Aim**

The aim of the NRHM is to reduce health inequalities by improving the availability of and access to better-quality healthcare, especially for people residing in rural areas (to reduce geographical inequality), for the poor (to reduce socioeconomic inequality), and for women and children (to reduce gender inequality).

**National Rural Health Mission’s Health Sector Plans**

**A. Health System Strengthening**

**Mobile Medical Units (MMUs)**

MMUs are a mechanism to provide outreach services in rural and remote areas. They are not meant for patient transfer. MMUs comprise one, two, or three vehicles, varying with the state. Where there is more than one vehicle, one vehicle is used for transport of medical and para-medical personnel, the second is used for carrying equipment/accessories and basic laboratory facilities, and the third vehicle carries diagnostic equipment such as X-Ray, ultrasound, and ECG machines and a generator. Each unit has one doctor, one nurse, one radiologist (if available), one lab technician, one pharmacist, and a helper and driver. The unit provides free medicines.

**Patient transport service**

Patient transport service (referral transport) involves patient transport ambulances operating under dial 108/102 ambulance services. The 102 services essentially consist of basic patient transport catering to the needs of pregnant women and children, though other categories are also benefiting and are not excluded. JSSK entitlements e.g. free transfer from home to facility, interfacility transfer in case of referral, and drop back for mother and children, are the key focus of the 102 service. The 108 service is predominantly an emergency response system, primarily designed to attend to critical care patients, trauma and accident victims, etc.

**Infrastructure strengthening**

Financial support is provided to states to strengthen the public health system, including upgrading existing or constructing new infrastructure. Under the NRHM, high-focus states can spend up to 33%, and other states up to 25%, of their NRHM funds on infrastructure.

**Human resources**

Financial support is provided to strengthen the health system, including engaging nurses, doctors, and specialists on a contractual basis, depending on an appraisal of requirements proposed by the states in their annual program implementation plans.

**Drugs and logistics**

States are being incentivized up to 5% of their total NRHM outlay to prepare policies and establish systems for free distribution of essential drugs, including drawing up an essential drug list (EDL), standard treatment protocols, robust procurement system etc., for free distribution of essential medicines in public health facilities. Various program components under the NRHM, such as Maternal Health, Child Health, Family Planning, Adolescent Health and National Disease Control Programs, prescribe specific drugs which are either centrally procured or for which funds are provided to States/UTs for decentralized procurement. Apart from program-specific drugs, funds are also provided for general drugs and supplies

**B. Maternal health care strategies**

**Janani Suraksha Yojna:** Launched in April 2005. Cash incentives are given to pregnant women for institutional deliveries. In low-performing states (LPSs), all pregnant women were beneficiaries, while in high-performing states (HPSs), pregnant women with a socioeconomic status below the poverty line were the beneficiaries. Schedule caste women are beneficiaries in both types of states.

**Janani Shishu Suraksha Karyakaram:** The initiative was launched in June 2011, and entitles all pregnant women delivering in public health institutions to absolutely free and no-expense delivery services, including caesarean section. The entitlements include free drugs and consumables, free diet up to 3 days during normal delivery and up to 7 days for C-section, free diagnostics, and free blood transfusion whenever required. This initiative also provides for free transport from home to the institution, or between facilities in case of a referral, and for drop back home. Similar entitlements have been put in place for all sick newborns accessing public health institutions for treatment till 30 days after birth. This has now been expanded to cover sick infants. The scheme aims to eliminate out-of-pocket expenses incurred by the pregnant women and sick newborns while accessing services at government health facilities.

**C. Child health care strategies and immunization**

Child health care strategies focused on providing newborn care units at the facility level through a facility-based neonatal care scheme, and skilled management of illnesses of children under five by training doctors and staff nurses and strengthening facilities under facility-based management of childhood illnesses. Medical officers and ANMs provided community-based management of ill newborns and children through integrated management of neonatal and childhood illnesses and home-based postnatal care. Malnutrition was targeted through approaches like infant and young child feeding practices and by establishing nutritional rehabilitation centers for severely malnourished children. Since mortality due to diarrhea and pneumonia is very high among children under 5, a targeted approach was used for these two diseases. Micronutrient supplementation was also provided. Immunization coverage was improved in remote and rural areas through provision of alternative vaccinators and increasing the number of outreach sessions.

**D. Communitization**

This included the provision of a female accredited social health activist (ASHA) for each village, who served as a link with the community health care delivery system. She belonged to the same village and had basic educational qualifications. She was given performance-based incentives for providing antenatal, postnatal, and child care. Village heads were involved by forming village health and sanitation committees. Maternal and child health services were provided by organizing village health and nutrition days each month in the village where antenatal care and immunization of children mainly took place.

**Appendix 2.**

**In-depth Interview/Focus Group Guide**

**Section 1: Informed consent**

**Section 2: Introduction to study (study information sheet provided to the participants)**

**Section 3: Extent of implementation of NRHM health sector plans for maternal and child health**

- What is your opinion on NRHM plans/schemes for maternal and child health?
- What do you think is the status of various schemes implemented to improve maternal and child health under the NRHM?

**Section 4: Status of maternal health strategies under NRHM implementation**

- What do you think about the implementation status of the existing maternal health strategies under the NRHM?
- Do you think that after the implementation of the NRHM plans, the maternal health has improved compared to the status before its implementation? If yes, how?
- Has the death rate among mothers decreased after the implementation of the NRHM? If yes, by how much?

**Section 5: Status of child health strategies under NRHM implementation**

- What do you think about the implementation status of the existing child health strategies under the NRHM?
- What is the immunization status among children? Are all children getting all vaccinations in time? Do you think the NRHM has helped in improving the immunization status of children?
- Do you think that neonatal (newborn) care has improved after the implementation of NRHM? Could you describe how?
- Do you think that after the implementation of the NRHM plans, child health has improved compared to the status before their implementation?

**Section 6: Accessibility, availability, and affordability of maternal and child health services**

- **Availability**: Do you think that the implementation of NRHM schemes and the maternal and child health services were able to reach rural areas, poor women and children, and disadvantaged groups?
- **Accessibility:** Do you think that there is improvement in the accessibility of maternal and child health services after the implementation of NRHM schemes in rural areas/ for poor women and children/ for disadvantaged groups?
- **Affordability:** In your opinion, are people satisfied with the existing NRHM schemes? Have NRHM schemes done anything to make maternal and child health services affordable for people, especially for people in rural areas and for poor families?

**Section 7: Status of maternal and child health inequalities after NRHM implementation**

- Do you think that health sector plans implemented under the NRHM influenced these inequalities in any way? If yes, how?
- Do you think that this inequality with respect to maternal and child health between rich and poor still exists?
- Has there been any improvement in these inequalities after the introduction of the NRHM?
- Do you think there are differences in health status between male and female children?

**Section 8:**

- Are people accepting the NRHM and its schemes?
- According to you, are people satisfied with existing NRHM schemes?
- Do you think there are barriers to implementing NRHM schemes that aim to reduce the geographic, socioeconomic, and gender health inequalities in the district?
- If yes, what in your opinion are the possible solutions to overcome these?
- What do you think is the key to success of the NRHM schemes?

**References**

1. Millenium development goals and beyond 2015. Available at <http://www.un.org/millenniumgoals/maternal.shtml>. Accessed on August 26, 2014. [↑](#endnote-ref-1)
2. Hota P. National Rural Health Mission. Indian J Pediatr 2006; 73:195-195. [↑](#endnote-ref-2)
3. Registrar General of India. Special Bulletin on Maternal Mortality in India. Sample Registration System 2010-12. Available at <http://www.censusindia.gov.in/vital_statistics/SRS_Bulletins/Final-MMR%20Bulletin-2007-09_070711.pdf>. Accessed on 16 July 2013. [↑](#endnote-ref-3)
4. Registrar General of India. Sample Registration System. Available at

   <http://censusindia.gov.in/Vital_Statistics/SRS/Sample_Registration_System.aspx>. Accessed on 15 July 2013. [↑](#endnote-ref-4)
5. [Ram](http://www.ncbi.nlm.nih.gov/pubmed/?term=Ram%20F%5Bauth%5D) F, [Singh](http://www.ncbi.nlm.nih.gov/pubmed/?term=Singh%20A%5Bauth%5D) A, Ram U. Human rights approach to maternal & child health: Has India fared well? Indian J Med Res 2013;137(4): 721–727. [↑](#endnote-ref-5)
6. [Singh](http://www.ncbi.nlm.nih.gov/pubmed/?term=Singh%20A%5Bauth%5D) A, [Pallikadavath](http://www.ncbi.nlm.nih.gov/pubmed/?term=Pallikadavath%20S%5Bauth%5D) S, [Ram](http://www.ncbi.nlm.nih.gov/pubmed/?term=Ram%20F%5Bauth%5D) F,  [Ogollah](http://www.ncbi.nlm.nih.gov/pubmed/?term=Ogollah%20R%5Bauth%5D) R. Inequalities in Advice Provided by Public Health Workers to Women during Antenatal Sessions in Rural India. PLoS One 2012; 7(9): e44931. doi:  [10.1371/journal.pone.0044931](http://dx.doi.org/10.1371%2Fjournal.pone.0044931) [↑](#endnote-ref-6)
7. [Singh A](http://www.ncbi.nlm.nih.gov/pubmed?term=Singh%20A%5BAuthor%5D&cauthor=true&cauthor_uid=22623976), [Padmadas SS](http://www.ncbi.nlm.nih.gov/pubmed?term=Padmadas%20SS%5BAuthor%5D&cauthor=true&cauthor_uid=22623976), [Mishra US](http://www.ncbi.nlm.nih.gov/pubmed?term=Mishra%20US%5BAuthor%5D&cauthor=true&cauthor_uid=22623976), [Pallikadavath S](http://www.ncbi.nlm.nih.gov/pubmed?term=Pallikadavath%20S%5BAuthor%5D&cauthor=true&cauthor_uid=22623976), [Johnson FA](http://www.ncbi.nlm.nih.gov/pubmed?term=Johnson%20FA%5BAuthor%5D&cauthor=true&cauthor_uid=22623976), [Matthews Z](http://www.ncbi.nlm.nih.gov/pubmed?term=Matthews%20Z%5BAuthor%5D&cauthor=true&cauthor_uid=22623976). Socio-economic inequalities in the use of postnatal care in India. PLoS One 2012; 7(5):e37037. doi: 10.1371/journal.pone.0037037. Epub 2012. [↑](#endnote-ref-7)
8. Nayar KR. Social exclusion, caste and health: A review based on the social determinants framework. Indian J Med Res 2007;126:355-363. [↑](#endnote-ref-8)
9. [Pathak PK](http://www.ncbi.nlm.nih.gov/pubmed?term=Pathak%20PK%5BAuthor%5D&cauthor=true&cauthor_uid=21798638), [Singh A](http://www.ncbi.nlm.nih.gov/pubmed?term=Singh%20A%5BAuthor%5D&cauthor=true&cauthor_uid=21798638): Trends in malnutrition among children in India: growing inequalities across different economic groups. Soc Sci Med 2011; 73(4):576-85. [↑](#endnote-ref-9)
10. Pradhan J, Arokiasamy P. Socioeconomic inequalities in child survival in India: A decomposition analysis. Health Policy. 2010. DOI: <http://dx.doi.org/10.1016/j.healthpol.2010.05.010> [↑](#endnote-ref-10)
11. Census 2011. [<http://censusindia.gov.in/2011census/censusinfodashboard/index.html>.] Accessed on Accessed on 16 July 2013. [↑](#endnote-ref-11)
12. Prusty RK, Kumar A. Socioeconomic dynamics of gender disparity in childhood immunization in India, 1992-2006. Plos One. 2014;15:9(8):e104598. doi: 10.1371/journal.pone.0104598. [↑](#endnote-ref-12)
13. Department of Economic and Statistical Analysis Haryana: Economic Survey of Haryana. Government of Haryana. 2012-13. Available at <http://web1.hry.nic.in/budget/Esurvey.pdf>. Accessed on 16 July 2013. [↑](#endnote-ref-13)
14. Registrar General of India. Sample Registration system. Matermal Mortality in India:1997-2003. Trends causes and risk factors. 2006:12-15. [↑](#endnote-ref-14)
15. Kaur M. Female Foeticide-A sociological perspective. The Journal of Family Welfare. 1998;39(1):40-43. [↑](#endnote-ref-15)
16. Sabu G, Dahiya M, Ranbir S. Female foeticide in rural Haryana. Economic and Political Weekly. 1998;33(32):2191-98. [↑](#endnote-ref-16)
17. Kumar R, Gupta M, Prinja S. Illness burden, care seeking and treatment cost among less than 2-year-olds in rural Haryana. Indian J Pediatr. 2014. Available at <http://www.ncbi.nlm.nih.gov/pubmed/24874811>. Accessed 1 December, 2014. [↑](#endnote-ref-17)
18. Sharma S. Child health and nutritional status of children: the role of sex differentials. Population Research Center. Institute of Economic Growth. Available at <http://www.iegindia.org/workpap/wp262.pdf>. Accessed 1 December 2014. [↑](#endnote-ref-18)
19. Child health program in India. Available at <http://mohfw.nic.in/WriteReadData/l892s/6342515027file14.pdf>. Accessed on 27 November 2014. [↑](#endnote-ref-19)
20. Hota P, Dobe M. National Rural Health Mission. Indian J Public Health 2005; 49(3):107-10. [↑](#endnote-ref-20)
21. National Health Mission. [[http://nrhm.gov.in/nhm/nrhm/nrhm-framework-for-implementation.html](http://nrhm.gov.in/nhm/nrhm/nrhm-framework-for-implementation.html).] Accessed on 16 January 2014. [↑](#endnote-ref-21)
22. Evaluation study of National Rural Health Mission (NRHM) in seven states. Programme Evaluation Organisation. Planning Commission. Government of India. Available at <http://planningcommission.nic.in/reports/peoreport/peoevalu/peo_2807.pdf>. Accessed on 29 June 2014. [↑](#endnote-ref-22)
23. Mukherjee S. A study on effectiveness of NRHM in terms of reach and social marketing initiatives in rural India. European Journal of Scientific Research 2010; 42(4):573. [↑](#endnote-ref-23)
24. [Pathak PK](http://www.ncbi.nlm.nih.gov/pubmed?term=Pathak%20PK%5BAuthor%5D&cauthor=true&cauthor_uid=21048964), [Singh A](http://www.ncbi.nlm.nih.gov/pubmed?term=Singh%20A%5BAuthor%5D&cauthor=true&cauthor_uid=21048964), [Subramanian SV](http://www.ncbi.nlm.nih.gov/pubmed?term=Subramanian%20SV%5BAuthor%5D&cauthor=true&cauthor_uid=21048964). Economic inequalities in maternal healthcare: prenatal care and skilled birth attendance in India, 1992-2006. PLoS One 2010; 27:5(10):e13593. doi: 10.1371/journal.pone.0013593. [↑](#endnote-ref-24)
25. [Pallikadavath S](http://www.ncbi.nlm.nih.gov/pubmed?term=Pallikadavath%20S%5BAuthor%5D&cauthor=true&cauthor_uid=23743004), [Singh A](http://www.ncbi.nlm.nih.gov/pubmed?term=Singh%20A%5BAuthor%5D&cauthor=true&cauthor_uid=23743004), [Ogollah R](http://www.ncbi.nlm.nih.gov/pubmed?term=Ogollah%20R%5BAuthor%5D&cauthor=true&cauthor_uid=23743004), [Dean T](http://www.ncbi.nlm.nih.gov/pubmed?term=Dean%20T%5BAuthor%5D&cauthor=true&cauthor_uid=23743004), [Stones W](http://www.ncbi.nlm.nih.gov/pubmed?term=Stones%20W%5BAuthor%5D&cauthor=true&cauthor_uid=23743004). Human resource inequalities at the base of India's public healthcare system. Health Place 2013; 23:26-32. [↑](#endnote-ref-25)
26. Leech NL, Onwuegbuzie AJ. A typology of mixed method research designs. Qual Quant 2009; 43:265-275. [↑](#endnote-ref-26)
27. Rural healthcare in System in India structure and current scenario. <http://mohfw.nic.in/Rural%20Health%20Care%20System%20in%20India.pdf>. (accessed 20 April 2014). [↑](#endnote-ref-27)
28. Panchayati Raj Ministry of Panchayati Raj. Government of India. <http://panchayat.gov.in/index.do?siteid=101&sitename=Government%20of%20India%20%3Cbr%3E%20Ministry%20of%20Panchayati%20Raj>. (accessed 20 April 2014). [↑](#endnote-ref-28)
29. International Institute of Population Sciences. District Level Household Surveys. Reproductive and Child Health Project. Ministry of Health and Family Welfare. New Delhi. India. Available at <http://www.rchiips.org/ARCH-1.html>. Accessed 10 July 2013. [↑](#endnote-ref-29)
30. Common Review Mission. Available at <http://nrhm.gov.in/monitoring/common-review-mission/1st-common-review-mission.html>]. Accessed 4 September, 2013. [↑](#endnote-ref-30)
31. Joint Review Mission. Available at http://nrhm.gov.in/monitoring/joint-review-mission.html. Accessed 4 September, 2013. [↑](#endnote-ref-31)
32. International Institute of Population Sciences. National Family Health Survey. Ministry of Health and Family Welfare. New Delhi. India. Available at <http://www.nfhsindia.org/factsheet.html>. Accessed 10 July 2013. [↑](#endnote-ref-32)
33. Goli S, Doshi R, Arokiasamy P. Pathways of economic inequalities in maternal and child health in Urban India: A decompostition analysis. Plos ONE 2013; 8(3): e58573. doi:10.1371/journal.pone.0058573 [↑](#endnote-ref-33)
